# Woman's Work in Medicine after the War

**Published:** 1916-09-02

**Authors:** 


### WOMAN'S WORK IN MEDICINE AFTER THE WAR.

Our contemporary, the *Evening Standard and St. James's Gazette*, recently called attention to the medical woman and her future. The subject is a vast one and at the present moment full of interest. It is one to which we have devoted many years of strenuous labour, and we can claim that we have placed her in a position in the nursing world which is one of the most huge benefits to humanity attained in recent years. But now, "while the world around lies overwhelmed," she who has for long "sailed between earth and heaven" must be moved to "disclaim those studies which possessed" her "in the dawn of life" and to find a fitting occupation among "the graver tasks of manhood." The words culled from what Akenside wrote in 1770 are true of aspiration in all time. They are free from the extravagant claims which threatened to wreck the equitable balance of life before the war, and, rather in fear than justice, now shine through some of the suggestions in the articles mentioned. We think, for instance, that too much is made of the normal variations in a woman's health, which have not been found to be a serious hindrance to her performance of her duty as a nurse. We admit with pleasure and thankfulness the help which her trained fingers and delicate manipulation have been in laboratory work, and the fervour with which she seeks an entrance into the province of the previously unknown. But we resent the limitations of her sphere should she desire to abandon the common lot of woman—"for an offering sacred to the powers who lent us gracious guidance."

The double rôle of parish nurse and doctor is surely rather an evolution from the parish nurse than a new sphere for the more highly educated woman who seeks to gain a foothold in a learned and honourable profession. Neither do we recognise that in assisting medical men to attend children and to dispense medicines she will find a suitable sphere. Women doctors should have as full an education and should pass as stringent examinations as medical men, unless we propose to create such a class of practitioners as was recognised in France during a portion of the last century and, to the credit of the profession, died a natural death. We prefer to hope that when this huge war is over the conditions of medical life will open a sphere for medical women which will occupy the talents of woman at her best. The allocation of large numbers of medical men to Army work, to the exclusion, for some years, of contact with the ills of daily life, will surely increase the number of those who choose a surgical career. The skill which such will attain under conditions which are not equalled in even the largest of our hospitals will be a possession not lightly to be set aside; and the numbers of cripples with which the country will be flooded will furnish problems for surgical treatment and mechanical device which are peculiarly the province of man. It may be that the training, and perhaps the crippling, of those on active service will tempt more men to seek employment in Public Health work than was formerly the case, and to the exclusion of women from a sphere in which in former times she found a suitable occupation. But the ailments of common life—those lesser things which have none of the urgency of war wounds and yet yearly swell the death roll of the country—the beginnings of disease upon which the early years of this century had begun to shed a brilliant light, will be more likely to attract women students than those whose minds have been stirred to struggle with injuries which are huge from their onset. The art of medicine will be in danger of suffering loss, and to its preservation woman may come as a helpful aid. We must not forget the attitude of the public.

It is certain that in the presence of overwhelming calamity those who have bearable ailments will hesitate to seek the advice of men who are already occupied with a graver task. To such the aid of a fully-trained and well-educated woman will be the greatest boon, and will often secure the preservation of health and life. Many of the cases which fall under her guidance will have relation to mental strain, the excessive production of hormones and chalones, and the altered nutrition of the ductless glands. This class of ailment is already obvious, and is likely to increase. A capable woman will not fail to estimate the beginnings of such diseases at their true value; her help will be sought without reserve, and her conclusions will save an important branch of medical science from slipping into abeyance. It is a huge subject, and only one of many. In all such the feminine temperament, which men know as a blessing in the home, will spread itself abroad—perhaps with the sacrifice of some hopes and many aspirations, but those women who are willing to come forward and meet the cost will realise a new phase in existence, and as W. E. Henley wrote, "Know life's a dream worth dreaming."

